# A Rare Case of Adenosine Deaminase tRNA-Specific 3 Mutation, Adrenal Insufficiency, and Rhabdomyolysis

**DOI:** 10.7759/cureus.19833

**Published:** 2021-11-23

**Authors:** Waheeb AlDhalaan, Faaezuddin Syed, Haroon A Javaid, Afaf AlSagheir, Sami Almustanyir

**Affiliations:** 1 Pediatric Endocrinology, King Fahad Medical City, Riyadh, SAU; 2 College of Medicine, Alfaisal University, Riyadh, SAU; 3 Pediatric Endocrinology, King Faisal Specialist Hospital and Research Centre, Riyadh, SAU

**Keywords:** electrolyte abnormalities, saudi arabia, adrenal insufficiency, rhabdomyolysis, adat3

## Abstract

Adenosine deaminase t-RNA-specific 3 (ADAT3) gene, present on chromosome 19, encodes for an enzyme responsible for deamination of adenosine to inosine. Individuals with ADAT3 mutation display microcephaly, dysmorphic features, neurological, behavioural, and endocrinal pathologies. ADAT3 mutation is a recognized cause of intellectual disability (ID) in Saudi Arabia, particularly amongst consanguineous families. Adrenal insufficiency (AI) is a life-threatening condition with variable clinical signs and symptoms, such as fatigue, nausea, vomiting, hypotension, hypoglycemia, and electrolyte imbalances. One very uncommon presentation of acute AI is rhabdomyolysis, a syndrome characterized by markedly elevated creatinine kinase (CK) levels, myoglobinuria, and muscle pain. We describe a case of an eight-year-old boy with ADAT3 mutation and growth hormone (GH) deficiency presenting with AI and rhabdomyolysis.

## Introduction

Adenosine deaminase t-RNA-specific 3 (ADAT3) mutation is a recognized cause of intellectual disability (ID) in Saudi Arabia, particularly amongst consanguineous families. Patients usually present with strabismus and tone abnormalities. Some endocrinopathies like growth failure have been implied to be part of manifestations of ADAT3-related ID syndrome [1]. The current literature does not report any cases of adrenal abnormalities associated with this mutation. Adrenal insufficiency (AI) is a life-threatening condition that presents with vague and undefined initial clinical symptoms [2]. One very uncommon presentation of acute AI is rhabdomyolysis [3]. To the extent of our literature review, our patient is the first pediatric case of autoimmune AI, presenting with rhabdomyolysis in the context of ADAT3 mutation.

## Case presentation

An eight-year-old boy was brought to the ED with a history of lethargy, decreased activity, muscle weakness, anorexia, and weight loss for a one-month duration. He was a known case of ADAT3 mutation and was taking Risperdal 0.5 mg daily for attention deficit hyperactivity disorder (ADHD) and Norditropin (growth hormone) two units subcutaneous daily for short stature. A physical examination done in ER displayed the patient to be lethargic and moderately dehydrated with the presence of hyperpigmentation in the buccal mucosa. The patient's heart rate, blood pressure, and blood glucose levels were 148 beats/min, 77/49 mmHg, and 2.4 mmol/L, respectively. A bolus of normal saline 20 ml/kg and ten boluses of dextrose 2 ml/kg were administered to the patient with continuous IV fluids. Serum lab investigations revealed the values listed in Table 1. Urine osmolarity was 325 mOsm/kg (300-800) with undetected myoglobin. Infection was ruled out as both culture and sensitivity of blood and urine were negative.

**Table 1:** Initial serum lab results.

Serum labs	Results
Urea	10.8 mmol/L (2.3-6.70)
Creatinine	46 umol/L (26-58)
Sodium (Na)	123 mmol/L (135-147)
Potassium (K)	7.2 mmol/L (3.5-5)
Glucose (random)	4.9 mmol/L (3.5-9)
Bicarbonate	18 mmol/L (22-31)
Osmolarity	260 mOsm/kg (285-295)
Creatine kinase (CK)	2983 U/L (24-195)

Management of hyperkalemia was started in the ER with a standardised protocol. Upon consultation with the endocrine team, one dose of hydrocortisone 50 mg IV was given and a plan of 15 mg IV every six hours was initiated. Repeated serum lab results after hydrocortisone administration are displayed in Table 2.

**Table 2:** Serum lab results after hydrocortisone administration.

Serum labs	Results
Adrenocorticotropic hormone (ACTH)	413 ng/L (5-60)
Cortisol	2699 nmol/L (High)
Renin	>500 mU/L (3-40 adult supine range)
Aldosterone	<4 ng/dL (Low)
Anti-adrenal antibody (21-hydroxylase)	1553 U/mL (Positive)

Four hours later, serums potassium (K) and sodium (Na) were 5.5 mmol/L and 126 mmol/L, respectively. Other endocrine workups included TSH, FT4, HBA1C, calcium, magnesium, phosphorus, and parathyroid hormone (PTH) which turned out to be normal. Autoimmune workup consisting of anti-thyroid peroxidase (TPO) antibodies, islet cell antibodies, antinuclear antibodies, and anti-parietal cell antibodies was also done, all of which were negative. The clinical finding and lab results were all consistent with primary AI due to autoimmune aetiology. The patient improved clinically, but blood pressure (BP) remained borderline, therefore, he was shifted to Pediatric ICU (PICU) for close monitoring. 

In PICU, his BP improved with revised serum lab readings of K 4 mmol/L and Na 138 mmol/L. The patient's medication regimen was shifted to 5 mg oral hydrocortisone to be given every eight hours and 0.05 mg oral fludrocortisone to be given daily. The patient was discharged home a couple of days later on a revised medication plan. 

One month after the discharge, the patient returned to the clinic with substantial improvement in his health. On inspection, he was active with decreased pigmentation. He exhibited a good appetite and gained almost 2 kg of weight. His labs were maintained at a normal range of Na 138 mmol/L, K 4.4 mmol/L, creatine kinase (CK) 113 U/L, and adrenocorticotropic hormone (ACTH) 24 ng/L.

## Discussion

ADAT3 mutation

ADAT3 mutation is a newly recognized cause of ID in Saudi Arabia, usually accompanied by strabismus and growth failure. Other features include microcephaly, short stature, and dysmorphic facial features, as well as neurological problems such as hypotonia, epilepsy, and behavioural problems such as ADHD [1]. ADAT3 gene, which is present on chromosome 19, encodes for an enzyme responsible for deamination of adenosine to inosine at the 34th position on a tRNA anticodon. It is a heterodimer that combines with ADAT2 to perform this function [4]. Several studies are trying to delineate the phenotype spectrum of ADAT3-related ID syndrome [1,5].

Adrenal insufficiency

Primary AI, also known as Addison’s disease, is a rare disorder, with an approximate prevalence of 93-140 per 1,000,000. It occurs due to the impaired synthesis of adrenocortical ‎hormones by the adrenal cortex. Autoimmune AI or autoimmune adrenalitis is the second most common cause of primary AI after congenital adrenal hyperplasia and accounts for around 15% of cases [6]. It is characterized by the presence of antibodies against antisteroidogenic enzymes (mainly 21-hydroxylase enzymes) and is often associated with the polyglandular autoimmune syndrome (type 2). Symptoms manifest only after around 90% of the cortex is destroyed [7]. The classical clinical presentation of an adrenal crisis is fatigue, nausea, vomiting, hypotension, hypoglycemia, and electrolyte imbalances (hyponatremia, hyperkalemia) [8]. However, early signs of the presentation can be quite variable and undefined. Markedly decreased BP along with the electrolyte abnormalities seen in our patient (hyponatremia, hyperkalemia, low bicarbonate, and hypo-osmolar serum) supports the diagnosis of AI. Apart from the mentioned electrolyte disturbances, our patient also presented with markedly elevated CK levels, which is explained by the coexisting rhabdomyolysis.

Rhabdomyolysis

Rhabdomyolysis is a syndrome of myonecrosis and associated spillage of contents into the circulation. Lab tests show markedly elevated CK levels. Myoglobinuria, muscle pain, and weakness are common symptoms. The aetiology can be traumatic or non-traumatic and includes immobilization, compression injuries, metabolic myopathies, infections, and drug-induced causes. Electrolyte disturbances, particularly hyponatremia, are rarely associated with rhabdomyolysis and are mostly due to primary polydipsia [9]. 

Rhabdomyolysis is rarely reported to occur along with acute AI. The pathophysiology of this association is still not fully understood. Interestingly, in most such cases, concomitant hyponatremia has also been reported. One study compared seven cases of adult patients with rhabdomyolysis associated with primary AI [3]. The majority showed hyponatremia and hyperkalemia, which is consistent with the electrolyte abnormalities displayed by our patient. One of these cases had a seizure before presentation, which was concluded to be the possible reason for high CK levels. Rhabdomyolysis is a well-known cause of acute kidney injury but only two of the seven cases demonstrated this. Only one other case of hyponatremic rhabdomyolysis with Addison’s disease has been reported in the pediatric age group [10]. This patient had autoimmune polyglandular syndrome (Carpenter syndrome) and reported hyponatremia of 120 mmol/L and hyperkalemia of 6.8 mmol/L,‎ which is quite similar to our patient's findings of 123 mmol/L and 7.2 mmol/L, respectively. Measuring CK levels in patients with Addison's disease to rule out accompanied rhabdomyolysis is suggested.

## Conclusions

This is the first case report of an ADAT3 mutation with growth hormone deficiency to present with an acquired AI and rhabdomyolysis in a pediatric age group. To prevent serious complications in other patients with ADAT3 mutation, further analysis is required to rule out any possible association with endocrinopathies such as AI.
